# The Effects of a DRO and Self-Monitoring Program on Prisoners in an Italian Prison

**DOI:** 10.1007/s40617-025-01045-0

**Published:** 2025-01-29

**Authors:** Valeria Pascale, Joshua K. Pritchard, Felice P. C. Iovino, Sara Bassani, Byron Wine

**Affiliations:** 1 Department of Legal Sciences, The University of Salerno, Salerno, Italy; 2 ABA for Disability, Salerno, Italy; 3 Factari LLC, Denver, CO USA; 4 The Faison Center, Richmond, VA USA

**Keywords:** Prison system, Italian prison treatment, Applied behavior analysis, DRO

## Abstract

This study examined the effectiveness of differential reinforcement of other behavior and self-monitoring in reducing challenging behavior among sixteen male inmates in an Italian prison. A multiple baseline design across groups was used to evaluate changes in property destruction, self-injury, and aggression. Results indicated a substantial reduction in these targeted behaviors across all participants. Social validity measures indicated improved inmate perception of their quality of life and interactions with others in the prison.

Applied behavior analysis (ABA) applies the science of behavior to socially significant issues. While perhaps currently best known for providing services to individuals with autism spectrum disorder, the science applies broadly to all aspects of social life involving human behavior. Heward et al. (2022) listed 350 applications of ABA, including many topics not commonly acknowledged as within the scope of ABA. Some notable examples from the paper include Alzheimer’s disease, aviation safety, exercise promotion, robbery prevention, and improving aspects of correctional facilities. This latter application of ABA is of interest to the current paper.

The issue of imprisonment is especially relevant in the United States as the country imprisons more of its population than any other democratic country, with 355 per 100,000 American residents imprisoned as of 2022 (Anderson et al. 2021). Additionally, the proportion of races imprisoned is not equitably distributed. For example, black Americans are 4.8 times more likely to be incarcerated than white Americans (Anderson et al. 2021). In addition to high rates and unequal distribution of imprisonment across races, there is evidence that the practice related to rehabilitating prisoners has many deficits. For example, the U.S. state prison systems reported that 82% of persons released from prison were arrested again at least once in a 10-year follow-up (Bureau of Justice Statistics, 2021). Certainly, there is room for improvement in the prison system.

Researchers have long demonstrated that ABA procedures effectively improve prisoners’ lives. Milan and McKee (1976) evaluated the effects of a token economy on completing a morning routine for a group of maximum-security prisoners. The token economy intervention was superior to praise alone or noncontingent token delivery. A follow-up analysis indicated that participating in the token economy did not decrease participants’ leisure time. Milan et al. (1979) made access to a popular token economy contingent upon completing an educational program. Most prisoners increased participation dramatically throughout the study. These types of interventions have proven successful, and some researchers have demonstrated that prisoners decreased problem behavior just to be able to participate in a token economy (Ellis, 1993).

Token economies have been adopted by the corrections literature with a number of demonstrations in the research. Gendreau et al. (2014) conducted a meta-analysis of the behavioral interventions in the corrections literature. The authors identified 29 studies conducted between 1965 and 2004. In all but one study, a token economy was the intervention of choice, and the authors found that these interventions were effective across several different target behaviors with an overall mean improvement of 54% across targeted behaviors. Most of these demonstrations are older, with many occurring during the 1970s. However, Gendreau stated that there is some evidence that incentive-based procedures, most often token economies, are reappearing in the literature.

More recently, researchers in ABA have demonstrated robust changes in drug abstinence and recovery in the context of criminal justice (Smith et al., 2004), as well as with sexual offenders (Reyes et al., 2006) among other areas, but relatively little research has been conducted on adult incarcerated individuals. Additionally, as in the corrections literature, many behavior analytic studies in adult correctional facilities are older, with much of the current literature focused on juvenile offenders in residential facilities (McDonald & Hanson, 2024).

Most interventions in the behavior analytic literature included in McDonald and Hanson’s (2024) review included multi-step token economies and point systems. However, recent literature also suggests that including self-monitoring has been effective in juvenile justice settings (Bush et al., 2023). Token economies and point systems are effective interventions but are complex and require significant resources, buy-in, and funds from senior leadership. It is unknown if simpler, easier-to-implement interventions, such as a differential reinforcement of other behavior (DRO) procedure, would be effective in prison.

The literature on applying behavior-analytic interventions to prisoners is limited and primarily consists of applying token economies, and much of the data are from the 1970s. Additionally, we know of no applications to prisons outside of the United States. The current investigation was initiated when a prison warden in Italy reached out to the lead author, who is a behavior analyst with a background in criminal justice, for assistance. The prison administrators requested a straightforward, easy-to-implement intervention that would enhance the prisoners’ and prison staff’s quality of life by decreasing problem behavior. The current study, therefore, was the first to implement a simplified behavior analytic intervention in a correctional facility outside of the United States.

## Method

### Participants and Setting

Sixteen male prisoners participated in the study. Participants ranged in age from 30 to 70 (*M* = 43). Participants’ demographic information was collected but not approved for dissemination. Exclusion criteria included individuals under the age of 18, documented mental neurocognitive or neurodevelopmental disorders, and individuals who had less than one year left on their sentences. As inmates serving sentences, all participants were convicted of one or more crimes. In order of prevalence, the most common convictions included theft, possession or trafficking of narcotics, robbery, receiving stolen goods, assault, battery, murder, property destruction, counterfeiting public seals or documents, and use of counterfeited seals or documents.

All individuals were incarcerated in a maximum-security detention treatment center throughout the study. The study did not modify the prison’s existing daily schedule. All inmates in the prison were allowed access to various activities from 8:00 am to 8:00 pm, including job skills, leisure, and meals. Leisure and job skills varied depending on the availability of instructors, and they included activities such as theater, yoga, and cooking, among others. All prisoners were locked in a cell between 8:00 pm and 8:00 am. Before data collection, all procedures were approved by the Dipartimento dell’amministrazione penitenziaria, Roma. Participants were recruited in person after approval. Informed consent was obtained from all participants prior to data collection.

### Measurement

The project was designed to target the most common and detrimental problem behaviors exhibited by the inmates. Potential targets were identified by asking the prison staff what were the most problematic in terms of frequency of occurrence, injuries to inmates, and disruption to the daily routine of the prison. The final list of problem behaviors tracked for all participants included property destruction, self-injury, and aggression toward others. Property destruction was defined as throwing, disassembling, or breaking any item. Common examples of property destruction included breaking lights and throwing furniture. Self-injury was defined as any attempt to harm their own person by any means. The most common examples of self-injury included cutting and burning with a lighter. Aggression toward others included any attempt to harm another person and most commonly involved striking, kicking, and attempting to choke other prisoners or staff members.

The prison staff collected data by recording whether or not each participant engaged in any target behaviors daily. The data were then aggregated per day and presented as the number of participants in each group who engaged in aggression, self-injury, and property destruction. Participants also collected data during the intervention but only the prison staff member’s data were graphed.

### Interobserver Agreement

A second independent observer collected data on 75% of the total days. The days evaluated were randomly selected across all conditions. The independent data collector shadowed the prison staff as the participants followed their daily schedule and recorded data without interacting with the prison staff or prisoners. Interobserver agreement (IOA) was completed by comparing the data sheets of the prison staff members and the secondary observer. An agreement was reached if the prison staff and the independent observer recorded the same number of problem behaviors across all participants for a given day. If there were any discrepancies between the count, then a disagreement for that day was recorded. IOA scores were calculated by dividing the number of days with agreements by the total number of agreements plus disagreements multiplied by 100. Total IOA was 100%.

### Experimental Design

We used a concurrent multiple baseline design to evaluate the effectiveness of the intervention. The participants were distributed randomly and evenly into four groups. Each group first experienced a baseline condition and was then sequentially exposed to the DRO and self-monitoring intervention.

### Procedure

#### Baseline

During baseline, there were no programmed changes to the daily routine in the prison while prison staff collected data on the targeted behaviors. The four groups of four participants remained in baseline until they were sequentially introduced to the intervention.

#### Differential Reinforcement of Other Behavior and Self-Monitoring

The differential reinforcement of other (DRO) behavior intervention and self-monitoring consisted of earning a token each day if the participants engaged in any behavior aside from those targeted (i.e., property destruction, aggression, or self-injury). A DRO procedure was selected because the prison warden approved the procedure, the intervention was relatively easy to implement, and past studies demonstrated the effectiveness of DRO procedures in reducing challenging behaviors with other populations (Wilder et al., 2023).

At the beginning of the intervention, each prisoner was presented with a contract describing the intervention procedures. Participants were given a token board that they kept in their cell, on which they were instructed to track their behavior. Tokens were blue stickers with a smiley face and were delivered daily by the prison staff after the prisoners returned to their cells for the evening. After the prisoners returned to their cells, the staff asked them to report whether they had engaged in any targeted responses during the day. The staff praised accurate responses and corrected prisoners who misreported. The tokens were then cashed in weekly on Saturdays.

Each week on Saturday, the participants had the opportunity to exchange seven tokens for extra edibles (e.g., pastries). If a participant did not earn seven tokens, the participant did not receive an edible pastry, and the rule about needing to earn a token each day was restated. If participants earned a token every day during the intervention, they could place one additional phone call to a family member and a small party.

#### Maintenance

The maintenance phase, which occurred when the intervention was discontinued, was identical to the baseline. It was conducted for all participants from day 41 to day 43.

#### Social Validity

A social validity assessment was delivered before data collection and at the end of the project. The lead author administered the social validity survey questions to each participant. The first author read the questions to each participant individually and recorded their answers. The questions were presented on a Likert scale from 1 to 5, in which the participants ranked how much they agreed with the statement (1 = not at all to 5 = completely agreed). The questions included and the results of the evaluations are presented in Table 1.

**Table 1:** Social validity questions and results from before and after the intervention

Questions	Before	After
Do you think your participation could improve your relationship	1.2	3.5
with others in the prison?		
Do you think that the absence of aggression and self-injury could	1.6	3.4
have a significant impact on your daily routine?		
Do you feel happy?	2.1	3.8
Do you think that the absence of property destruction could have	2.8	2.3
a significant impact on your daily routine?		
Would you like to keep the intervention in place permanently?	1.0	3.8

## Results

**Fig. 1:**
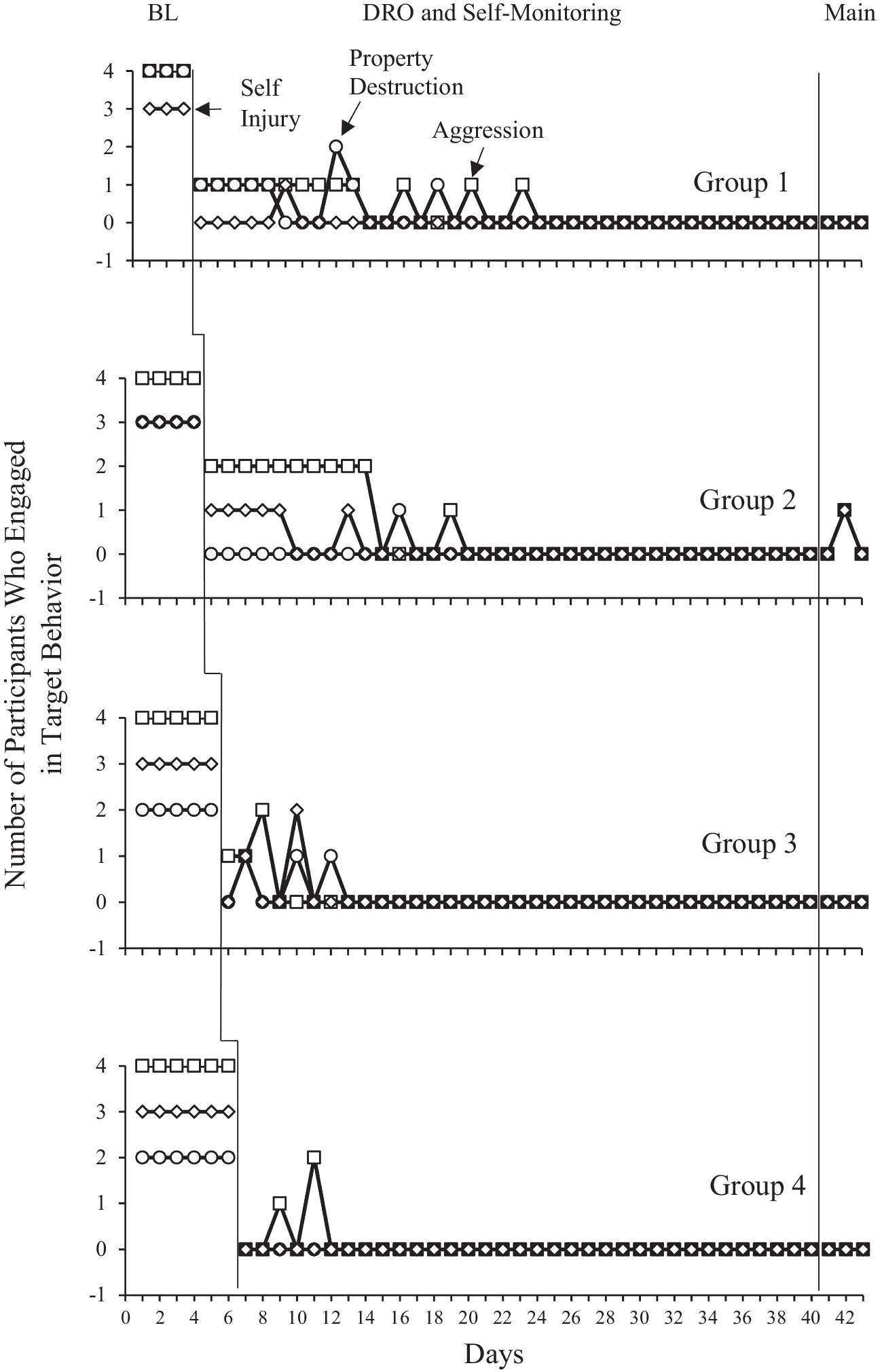
Number of participants who engaged in targeted behaviors per day during baseline, intervention, and maintenance phases

Figure 1 presents the results for each group. Group 1’s baseline (first tier) lasted three days, during which all four participants engaged in aggression and property destruction, and three of the four engaged in self-injurious behavior at least once per day. Once the intervention was introduced, the number of prisoners engaging in each target behavior dropped substantially. By the 24th day, none of the participants engaged in any targeted behaviors for the remainder of the intervention, and no participants engaged in any targeted behaviors during the maintenance phase.

Group 2’s baseline (second tier) lasted four days. During this time, all four prisoners engaged in aggression each day, and three participants engaged in property destruction and self-injurious behavior each day. After the intervention was introduced, the number of participants who engaged in targeted behavior decreased immediately. This downward trend continued, with no targeted behavior exhibited by participants after the 20th day. During the maintenance phase, one participant engaged in property destruction and self-injury on day 42.

Group 3’s baseline (third tier) lasted for five days. In baseline, all four participants in this group exhibited aggression in each of the first five days of baseline. Two participants engaged daily in property destruction, and three participants engaged in self-injury daily during baseline. Once the intervention was implemented, the target behavior dropped immediately, and by day 13, none of the participants engaged in any targeted behavior. No participants engaged in any targeted behaviors during the maintenance phase.

Group 4’s baseline (fourth tier) lasted for six days. In baseline, all of the participants engaged in aggressive behavior each day. Two participants engaged in property destruction daily, and three participants engaged in self-injury daily. After the intervention was implemented, all targeted behaviors dropped immediately and were no longer observed after day 12. No participants engaged in any targeted behaviors during the maintenance phase. After the intervention, the social validity assessments demonstrated an increase in all questions except question 3, which dealt with the importance of property destruction. Table 1 presents these data. 

## Discussion

This was the first study in Italy to use an intervention from the ABA literature in a correctional facility context. The data suggest that the DRO and self-monitoring intervention effectively reduced the prevalence of the targeted behavior across all sixteen prisoners. The effects were quickly realized and maintained even after the intervention was withdrawn. Additionally, the prison staff had no experience with ABA but were able to implement the data collection procedures after a few short meetings. The intervention was simple, cost-effective, and generally well-liked by the participants.

The program made two significant improvements in the prison context. First, the prisoners generally scored the program as increasing their happiness and improving their relationships with others. Improving the lives of incarcerated individuals is important in and of its own right given research showing that the interruption of everyday life inherent in prison has serious consequences for prisoner mental health (Armour, 2012). The intervention also decreased aggression and self-harm. Decreasing these behaviors is important not only to other prisoners who are targeted but to prisoners who are not intended victims but have to witness the acts (Novisky & Peralta, 2020).

As an applied study, the current investigation is not without limitations. First, although a multiple baseline design was implemented, there was only one overlapping data point between each cohort of participants moving into the intervention condition. This was done at the request of the prison administration to decrease time spent in baseline. Additionally, the participants were all housed in the same location, and they could have discussed the intervention. Given that behavior change was immediate between baseline and intervention for most participants this suggests that they had discussed the intervention or had generated rules about the intervention from the informed consent process and so the exact mechanism for change cannot be identified. Moreover, since the DRO and self-monitoring components were implemented simultaneously it is not possible to determine what component was responsible for the change in behavior. Finally, many of the participants had several years remaining on their term of incarceration, and it is unclear if the intervention would have remained effective over time given the relatively short duration of the study.

There are many avenues for future research. Future research in this area should explore different types and scopes of interventions leveraged within a prison to enhance quality of life for prisoners and staff. This is especially important in terms of cost and effort required to implement. As more technologies for this population are developed, it will be important to examine which components of the treatment packages function as agents of change. One initial step in this direction could include a comparison of the self-management and externally delivered tokens in this package. Additionally, despite being commonly referred to as a reinforcement paradigm, DRO could also be conceptualized as a negative punishment paradigm. That is, it could be that problem behavior decreased because the loss of an appetitive stimulus was available contingent upon problem behavior. Research into how DRO or other programs based upon positive reinforcement (i.e., token economy) could influence how behavior change programs should be constructed, in terms of social validity and effectiveness, in detention settings.

It is currently unknown if behavior analytic strategies could have an impact on recidivism rates; this could be especially interesting in settings where prisoners are encouraged to attend school or other development programs to better prepare them to reintegrate into society after release. If a token economy or some other intervention encouraged prisoners to complete training programs that increased access to employment after their prison term was completed, it is possible that these interventions could impact recidivism by decreasing the likelihood of the individuals reoffending. Finally, the participants in this study made it clear in informal interviews that they desired a means to earn social time with external persons (e.g. primary investigator), but this was not implemented or examined empirically and so extra opportunities for extra social contact could be evaluated in future investigations.

This study is a rejoinder to earlier work in ABA. The interventions developed were effective across countries and may benefit prison systems. We hope this study will encourage others to attempt work in this underserved population. While relatively few behavior analysts work in correctional facilities, some clients are in situations where they have relatively little say in the structure of their environment (e.g., public school or residential facilities). Data concerning interventions that are effective, both in terms of behavior change and social validity, when clients must attend non-preferred or restrictive settings is beneficial for behavior analysts.

## Declarations

### Author note

All procedures performed in studies involving human participants were in accordance with the ethical standards approved by the University of Salerno research department.
