# Correction to: The Effects of a DRO and Self-Monitoring Program on Prisoners in an Italian Prison

**DOI:** 10.1007/s40617-026-01245-2

**Published:** 2026-09-25

**Authors:** Valeria Pascale, Joshua K. Pritchard, Felice P. C. Iovino, Sara Bassani, Byron Wine

**Affiliations:** 1 Department of Legal Sciences, The University of Salerno, Salerno, Italy; 2 ABA for Disability, Salerno, Italy; 3 Factari LLC, Denver, CO USA; 4 The Faison Center, Richmond, VA USA

**Correction to: Behavior Analysis in Practice**

**https://doi.org/10.1007/s40617-025-01045-0**

This article was updated to correct the author list. On the original publication Felice P. C. Iovino was listed as Felice P. C. and Iovino.

